# Internet and social network users’ profiles in Renal Transplant Recipients in France

**DOI:** 10.1186/s12882-017-0670-y

**Published:** 2017-08-03

**Authors:** Yosra Mouelhi, Marine Alessandrini, Vanessa Pauly, Bertrand Dussol, Stéphanie Gentile

**Affiliations:** 1 Laboratoire de Santé Publique, Faculté de Médecine, Université Aix-Marseille, 3279 Marseille, EA France; 2 Service Santé Publique et Information Médicale, CHU Marseille, Marseille, France; 3 Centre de Néphrologie et de Transplantation Rénale, CHU Marseille, Marseille, France

**Keywords:** Characteristics, Inclusion, Internet, Profiles, Renal transplant recipient, Social networks users

## Abstract

**Background:**

The use of the Internet for searching and sharing health information and for health care interactions may have a great potential for Renal Transplant Recipients (RTR). This study aims to determine the characteristics associated with Internet and social network use in a representative sample of RTR at the time of their inclusion in the study.

**Methods:**

Data of this cross-sectional design is retrieved from a longitudinal study conducted in five French kidney transplant centers in 2011, and included Renal Transplant Recipients aged 18 years with a functioning graft for at least 1 year. Measures include demographic characteristics (age, gender, level of education, employment status, living arrangement, having children, invalidity and monthly incomes in the household), psycho-social characteristics measured by the perceived social support questionnaire, and medical characteristics (previous dialysis treatment, duration since transplantation, graft rejection episodes, chronic graft dysfunction, health status and comorbidities: neoplasia for the current transplant, hypertension, diabetes mellitus, smoking status, BMI > 30 kg/m^2^ and Charlson Comorbidity Index (CCI)). Polytomous linear regression analysis was performed to describe the Internet and social network users’ profiles, using lack of Internet access as the comparison category.

**Results:**

Among the 1416 RTR participating in the study, 20.1% had no Internet access in the household, 29.4% connected to social networks and 50.5% were not connected to social networks. Patients who connected the most to the Internet and social networks were younger, male, without children, employed, with high monthly incomes in the household, without hypertension and having felt a need for an informative or an esteem support.

**Conclusion:**

In our study, the majority of RTR were actively using Internet and social networks. Renal transplant units should develop flexible and Web-based sources related to transplant information, which will allow a rapid adaptation to changes in prevalent practice, improve the health of the patients and reflect their preferences.

## Background

The rapid growth of Internet technology and Internet users over the past 2 years has created a new virtual public meeting place and changed the way people work, play, learn, and communicate [1].

Daily, more than 12.5 million health-related computer searches are conducted on the World Wide Web (WWW) [2]. In France, as in other European countries, the number of computer users who have access to the Internet has increased. In 2016, according to the agency ‘*We Are Social’,* the penetration rate of the French internet users reached 86% instead of 84% in 2015, while the penetration rate of the French social networks users attained 50% [3].

The Internet has become a popular communication tool that plays a key role in patient-centered care [4]. It has been used as a source of health information since the early 1990s [5]. Websites on the Internet are increasingly used by patients and caregivers as a source of medical information for several diseases [6], as it provides a widely accessible modality for meeting psycho-educational, information, and resource needs for patients. This provides the potential opportunities for patients suffering from chronic diseases to become well-informed and to take an active part in their own treatment process. Two British surveys have recently shown a gradual increase in public interest in the use of the Internet for health information [7, 8]. The Pew Research Center’s Internet & American Life Project describes that 53% of smartphone owners have used their phone to look for medical information [9].

Furthermore, the use of the Internet for health care interactions may represent a necessity for patients with rare diseases to better manage their complex health needs [10]. Sharing networks for health related information, such as symptoms, treatments, prescriptions, and diet related information, could thus be beneficial for individuals and their social networks [6, 11]. Indeed, patients with serious illnesses can learn from other individuals with similar conditions by connecting through the Internet. Fox and Jones have found that 41% of e-patients have read someone else’s experience or commentary about their health on a Web-based news group, website, or blog [12]. According to recent Pew Internet report, about 23% of Internet users in the US have followed their friend’s personal health experience in the past year, with a 3% increase compared to 2010 [13]. A recent survey among Italian families of patients with rare diseases showed that parents frequently participate in forums and online communities, where they mostly share information about their child’s disease [14]. Other surveys have also shown that sharing health related knowledge online has become an important habit among people [15, 16].

Because of the complexity of renal transplantation, its risks and attendant ethical considerations, providing new opportunities, establishing guidelines and opening ongoing access to information are essential for patients’ understanding after transplantation as a potential psychosocial intervention.

In this context, five French kidney centers were interested to analyze the profiles and the ways in which patients use this technology for social support after renal transplantation. This paper aims to determine the characteristics associated with Internet and social network use in a representative sample of Renal Transplant Recipient (RTR). Furthermore, the outcomes could be used in making strategies for the patients to develop Internet sources and to support patient management in the modern era of E-technology.

## Methods

### Study design

Data of this cross-sectional design is retrieved from a longitudinal study conducted in five French kidney transplant centers in 2011, and included all RTR aged 18 years with a functioning graft for at least 1 year. This report analyzes only data of inclusion.

### Data collection and measures

At the time of inclusion, data were collected directly from self-administered questionnaires completed by patients agreeing to participate, including demographic, psycho-social and medical characteristics.

The dependent variable was Internet status. Three categories of Internet status were analyzed: no Internet access, Internet access without social networks use and Internet access with social network use.

Concerning the independent variables, only the following characteristics were analyzed in this report:

***➢ Demographic characteristics:*** Age, gender, level of education, employment status (unemployed versus employed), living arrangement (living alone versus other status), having children, invalidity and monthly incomes in the household.

***➢ Psycho-social characteristics:*** measured by the perceived social support questionnaire [17] composed of four main subscales: esteem, financial, informative and emotional supports.

***➢ Medical characteristics:*** previous dialysis treatment (before transplantation), duration since transplantation, graft rejection episodes (none versus one at least), chronic graft dysfunction, health status and comorbidities: neoplasia for the current transplant, hypertension, diabetes mellitus, smoking status, BMI > 30 kg/m^2^ and Charlson Comorbidity Index (CCI).

### Ethics approval and consent to participate

The study methodology was approved by the local Institutional Review Board: “Comité Consultatif sur le Traitement de l’Information en matière de Recherche dans le domaine de la santé” (CCTIRS) n°12–726 and the “Commission Nationale de l’Informatique et des Libertés” (CNIL) n°1,639,707, thus ensuring the confidentiality of all the collected information. All patients agreeing to participate signed a written informed consent before their inclusion in the study.

### Data analysis

Data were analyzed on SPSS® Statistics 20 software and included both descriptive and multivariate analyses.

First, to compare the three groups (no Internet access, Internet access with or without social network use) according to the different characteristics, we used Student test for quantitative variables and chi-square test for categorical ones.

Then, to determine characteristics associated with the use of internet and social networks (the dependent variable, categorical with 3 categories), we performed the Polytomous Logistic Regression model (PLR) [18], using no Internet access as the comparison category. We introduced all the variables into the multivariate model with a *p*-value <0.20 in the univariate model, then we performed a backward elimination procedure with the purpose of conserving only the variables with *p*-value < 0.05 in the final model. The adjusted odd ratio (OR) values with their 95% confidence interval were performed.

## Results

### Description of Internet statuses

Among the 1416 RTR participating in the study, 20.1% (*n* = 285) had no Internet access in the household at the time of inclusion and nearly 80% (*n* = 1131) of RTR had access to the Internet. Among them, 29.4% (*n* = 417) connected to social networks while 50.5% (*n* = 714) did not.

Table 1 shows the univariate analysis. The variables that presented a statistically significant association (*p* < 0.05) with Internet access and social network use were: younger age (47.2 ± 13.6 years), male gender (58%), employment status (59.4%), living alone (75.1%), having children (63.9%), high monthly incomes in the household (34.2%), having felt a need for an esteem support (65.9%), having felt a need for an informative support (57.9%), without one acute rejection (79.1%), without neoplasia (86.3%), without diabetes mellitus (85.6%), non-smoking patients (79.7%), with hypertension (72.4%) and Charlson Comorbidity Index between 1 and 2 comorbidities (41.7%) (Table 1).

**Table 1:** Patients characteristics by Internet Status (*N* = 1416)

	No Internet access (*n* = 285)	Internet access without social network use (*n* = 714)	Internet access with social network use (*n* = 417)	Total	*P* univariate
Demographic characteristics					
Age					
* Mean ± SD* ^a^	62.7 ± 10.6	57.7 ± 11.2	47.2 ± 13.6	55.6 ± 13.2	<0.001
* Range*	20.5–85.9	23.5–83.5	18.8–78.9	18.8–85.9	
Gender					
* Male*	161 (56.5)	465 (65.1)	242 (58.0)	868 (61.3)	0.01
* Female*	124 (43.5)	249 (34.9)	175 (42.0)	548 (38.7)	
Employment status					
*Unemployed*	256 (89.8)	444 (62.2)	169 (40.6)	869 (61.4)	<0.001
*Employed*	29 (10.2)	270 (37.8)	247 (59.4)	546 (38.6)	
Living arrangement					
*Alone*	192 (67.6)	587 (82.2)	313 (75.1)	1092 (77.2)	<0.001
*Not alone*	92 (32.4)	127 (17.8)	104 (24.9)	323 (22.8)	
Children					
* No children*	84 (29.7)	155 (21.8)	150 (36.1)	389 (27.6)	<0.001
*Having children*	199 (70.3)	557 (78.2)	266 (63.9)	1022 (72.4)	
Monthly incomes (€)					
*<739*	40 (15.4)	38 5.6)	22 (5.6)	100 (7.5)	<0.001
*740–1200*	89 (34.4)	94 (13.8)	72 (18.4)	255 (19.2)	
*1201–2200*	89 (34.4)	204 (30.0)	123 (31.4)	416 (31.3)	
*2201–4400*	33 (12.7)	257 (37.8)	134 (34.2)	424 (31.9)	
*>4000*	8 (3.1)	87 (12.8)	41 (10.5)	136 (10.2)	
Perceived social support					
Having felt a need for an esteem support					
* No*	129 (45.4)	280 (39.2)	142 (34.1)	551 (38.9)	0.01
* Yes*	155 (54.6)	434 (60.8)	275 (65.9)	864 (61.1)	
Having felt a need for an informative support					
* No*	193 (68.0)	420 (59.1)	241 (57.9)	854 (60.5)	0.01
* Yes*	91 (32.0)	291 (40.9)	175 (42.1)	557 (39.5)	
Medical characteristics					
At least one acute rejection episode					
* No*	238 (86.5)	614 (87.3)	321 (79.1)	1173 (84.8)	0.001
* Yes*	37 (13.5)	89 (12.7)	85 (20.9)	211 (15.2)	
Health status and comorbidities					
Neoplasia					
* No*	208 (75.6)	540 (76.9)	354 (86.3)	1102 (79.5)	<0.001
* Yes*	67 (24.4)	162 (23.1)	56 (13.7)	285 (20.5)	
Hypertension					
* No*	29 (10.5)	112 (15.9)	113 (27.6)	254 (18.3)	<0.001
* Yes*	248 (89.5)	592 (84.1)	297 (72.4)	1137 (81.7)	
Diabetes mellitus					
* No*	203 (73.3)	583 (82.8)	351 (85.6)	1137 (81.7)	<0.001
* Yes*	74 (26.7)	121 (17.2)	59 (14.4)	254 (18.3)	
Smoking patients					
* No*	239 (88.5)	597 (87.0)	322 (79.7)	1158 (85.1)	0.001
* Yes*	31 (11.5)	89 (13.0)	82 (20.3)	202 (14.9)	
Charlson Comorbidity Index					
* 1–2 comorbidities*	23 (8.1)	141 (19.7)	174 (41.7)	338 (23.9)	<0.001
* 3–4 comorbidities*	94 (33.0)	310 (43.4)	159 (38.1)	563 (39.8)	
* >5 comorbidities*	168 (58.9)	263 (36.8)	84 (20.1)	515 (36.4)	

Percentage in column, ^a^ *SD* Standard Deviation

### Characteristics associated with internet and social network use

The polytomous logistic regression model was used to determine the characteristics associated with Internet and social network use.

The reference category in the model was “no Internet access” (*n* = 285), provided in Table 2. We compared this category to patients with Internet access and social network use (*n* = 417) and to patients without social network use (*n* = 714). The variables in the model included demographic, psycho-social and medical variables.

**Table 2:** Polytomous logistic regression: characteristics associated with Internet and social network status (reference: no Internet access)

Variables	Internet access without social network use (*n* = 714) Versus no Internet access (*n* = 285)		Internet access with social network use (*n* = 417) Versus no Internet access (*n* = 285)	
	Adjusted OR* (95% CI*)	*P*	Adjusted OR* (95% CI*)	*P*
Age	0.9 [0.9;1.01]	0.06	0.8 [0.8;0.9]	<0.001
Gender (Reference = female)				
Male	1.4 [1,03–2,02]	0.04	1.05 [0.7–1.5]	0.7
Children (Reference = no children)				
Having children	0.5 [0.3;0.7]	0.002	0.4 [0.3;0.7]	0.003
Employment status (Reference = employed)				
Unemployed	0.4 [0.2;0.6]	0.001	0.3 [0.2;0.6]	<0.001
Monthly incomes (Reference= >4400 €)				
<739	0.08 [0.03;0.2]	<0.001	0.05 [0.01;0.1]	<0.001
740–1200	0.1 [0.04;0.2]	<0.001	0.08 [0.03;0.2]	<0.001
1201–2200	0.2 [0.1;0.5]	0.001	0.2 [0.08;0.5]	0.001
2201–4400	0.7 [0.3;1.7]	0.4	0.7 [0.2;1.9]	0.5
Esteem support (Reference = yes)				
No	0.9 [0.6;1.3]	0.6	0.6 [0.3;0.9]	0.04
Informative support (Reference = yes)				
No	0.6 [0.4;0.9]	0.04	0.9 [0.5;1.4]	0.6
Hypertension (Reference = yes)				
No	1.5 [0.9;2.6]	0.08	2.1 [1.1;3.6]	0.009
Charlson Comorbidity Index (Reference= > 5 comorbidities)				
1–2 comorbidities	0.2 [1.06;5.3]	0.03	0.9 [0.3;2.1]	0.8
3–4 comorbidities	1.7 [1.1;2.6]	0.01	1.2 [0.7;2.01]	0.4

*Abbreviations*: *Reference* No Internet access, *OR Odds Ratio, *CI Confidence Interval

#### Characteristics associated with internet access without social network use

There were 714 RTR who reported that they had access to the Internet but without social network use. Compared to participants without Internet access, factors significantly associated with this category were: male gender, having no children, employed status, high monthly incomes in the household (>4400 €), having felt a need for an informative support and a high score of the Charlson comorbidity Index.

#### Characteristics associated with internet access and social network use

Social network use characterized 417 participants in the study. Compared to patients without Internet access, social network users were younger, without children, with high monthly incomes in the household (>4400€) and having felt a need for an esteem support. Furthermore, patients without hypertension used social networks the most.

## Discussion

This report analyzes the characteristics of the participants using both the Internet and social networks in a representative sample of 1416 Renal Transplant Recipients (RTR). To our knowledge, there is no study which analyzed the profile of Internet and social network users after renal transplantation, and few studies were carried out in chronic diseases.

Our results showed that Internet is a popular source used by the majority of RTR (79.9%). This was in accordance with a survey from Scotland which showed that 87% of kidney-transplanted patients had access to the Internet [19]. Moreover, nearly 30% of our patients connected to social networks. Several studies showed that social networks are popular among people affected with chronic diseases, as they provide support in chronic diseases, especially with Facebook and blogs [20–22].

In accordance with literature [19, 23, 24], younger age, male gender, having no children, employment status and high monthly incomes in the household, were associated with Internet access and social network use. However, a recent study found that females use Internet the most [25].

Our results also showed that there is a relationship between social support, Internet and social networks. Patients which needed an informative support had the most access to the Internet. This result was in accordance with the Scottish survey: among the 87% of RTR having access to the Internet, 70% used it for seeking health information and 90% looked up information on transplantation [19]. It is possible that Internet may allow those patients to easily seek information about their transplant and to be involved in their own health care. Furthermore, in our study, patients which needed an esteem support used social networks the most. Indeed, Coiera E [26] indicated that social networks can directly support disease management by creating online spaces, which give more confidence to patients and encourage them to interact with clinicians and share experiences with other patients. For example, cancer patients use Twitter to discuss treatments and provide psychological support [27] and online engagement seems to correlate with lower levels of self-reported stress and depression [28]. Hwang et al. [29] reported that encouragement and shared experience were important social support features of social media sites.

Moreover, RTR with several comorbidities connected the most to the Internet and social networks. It is possible that these patients use Internet to compensate for a lack of information from the healthcare system. For patients who have difficulties understanding information provided by physicians, the Internet may be a useful complement for secondary prevention, especially to better understand health problems or to enhance therapeutic compliance [30]. In contrast, patients with hypertension connected less to social networks, which suggests a higher awareness for them. Several studies have shown that a worse social network leads to higher blood pressure levels [31, 32]; a systematic review affiliated with Mayo Clinic has even recommended that the lack of social network use is a risk factor for subsequent morbidity and mortality after a Myocardial Infarction [33].

Consequently, our findings suggest that RTR may need more psychological interventions aiming to provide information about their medical care. It is possible that patients after renal transplantation are continuously faced with adaptive tasks [34], which explains that the availability of social support from the personal network could be one of the factors supporting an individual’s adjustment to a chronic disease [35, 36]. This could help them to deal with their transplant and reduce several mental problems such as stress and anxiety.

The Internet and social media, in recent years, have significantly changed the health communication landscape, according to their accessibility, speed and anonymity [11, 37]. They have rapidly become an important source of health information, public education, organizational promotion, patient care, patient education and advocacy regarding public health issues [38].

Several recent studies showed that Internet interventions are effective for improving health care and widening access to health information to various population groups, regardless of age, education, race or ethnicity, and locality, compared to traditional communication methods [38–41]. A review of 24 randomized studies found that Internet-based interventions that combine health information with social, decision, or behavior change support, has significantly changed patient knowledge, perceived social support, key behavioral and clinical outcomes compared to non-web-based control programs [42]. Other studies have shown that electronic communication may improve patient satisfaction by increasing the time spent communicating with and having questions answered by their physicians [38]. A survey of patients found that 56% of them wanted their health care professionals to use social media for appointments, diagnostic test results, and for answering general questions [43]. Patients who did not use Internet and social networks said they would start using them if they could connect with their health care provider.

Social media can also improve patients’ access to health care information and other educational resources [44], they can join virtual communities and connect with others affected by similar conditions. For example, the social networking site PatientsLikeMe provides a way for patients to access information, suggestions, and support from other people who are suffering from the same disease or condition. Facebook groups also frequently focus on specific health conditions and actively engage in peer-to-peer support for individuals [11].

Furthermore, social media can provide health care professionals with tools to share information, debate health care policy and practice issues, share cases and ideas and to consult colleagues regarding patient issues [37, 45, 46]. These professionals also use social media, including Twitter and Facebook, to enhance communication with patients [45, 47]. Househ M [46] found that physicians have begun to develop an interest in interacting with patients online, with the purpose of providing patient education and health monitoring, and encouraging behavioral changes and drug adherence, with the hope that these efforts will lead to “better education, increased compliance, and better outcomes”.

In addition to their efficacy, one of the most commonly cited reasons to implement Internet and social media interventions is reducing health services and delivery costs [48]. The ability to access health information at home may reduce the cost of having to travel to and from a health care service. Few studies have examined issues related to cost of Internet-based interventions and comparing them to costs for traditional delivery mechanisms. They concluded that the Internet-based programs were more cost-efficacious than face-to-face treatments delivered by a therapist [49–51].

Although the cost-efficacy of Internet and social media for health information have been established, it is becoming difficult to distinguish which resources or websites are accurate or appropriate for patients [52]. The main limitation of health information found on social media and other online sources is the lack of quality and reliability. Authors found that social media sites for medical information are often unknown or identified by limited information. Studies about liver and kidney transplants have shown that the educational material available on the Internet is of poor quality and requires rigorous input from health care professionals [53–55], and none of the Internet tools has proven credibility to reliably judge transplant Web sites [56].

## Conclusion

In this study, we found that patients after renal transplantation actively use the Internet and social networks. By opening authenticated Websites and blogs related to those patients, nephrologists can recommend them to their patients, which can help the latter to improve their healthcare and to save time. This constructive contribution by nephrologists may lead to a more efficient use of social media. Renal transplant units should develop flexible and Web-based sources related to transplant information, which will allow a rapid adaptation to changes in prevalent practice, improve the health of the patients and reflect their preferences.

## Abbreviations

BMI: Body mass index
CCI: Charlson comorbidity index
CI: Confidence interval
CKD: Chronic kidney disease
OR: Odds ratio
PLR: Polytomous linear regression
RTR: Renal transplant recipient
SD: Standard deviation
